# Tatooing the Cornea

**Published:** 1873-09

**Authors:** Henry W. Williams


### Tatooing the Cornea.

By Henry W. Williams, M. D.

"The removal of conspicuous blemishes of the cornea by a safe and nearly painless process of tatooing deserves to be regarded as one of the most valuable recent improvements in ophthalmic surgery.

Although known to the ancients from the time of Galen, it had quite fallen into oblivion; and, though not having the importance of those capital operations which preserve or restore the sight, it yet confers a great benefit upon patients by ridding them of what is often a marked or even a repulsive deformity.

The central part of the leucoma may be tinted with india-ink, to represent the pupil, and the marginal portions of the opacity with brown, blue, or other water colors, to correspond with the tint of the iris in the other, healthy eye. The ink should be rubbed up with water to the consistence of a thick paste, and may then be preserved for use, in a fluid condition, by adding glycerine.

The process of pricking in should be begun at the lower part of the space it is intended to cover. Wecker uses a spring elevator, to keep the lids separated; but this is more complained of than the operation itself, and is not necessary. It is sufficient to press the lids open with the thumb and finger, which may be covered, as suggested by Warlomont, with a bit of rag, to prevent slipping and to absorb the tears. A cataract needle, an instrument composed of several very fine sewing needles bound together and inserted into a handle, or any delicate pointed instrument, may serve for the tatooing. A little of the thick pigment being taken up on the needle and placed upon the cornea, a large number of punctures are rapidly made, through the epithelial layer only, over the surface it is proposed to color, and the pigment is then gently rubbed into the punctures with the end of the finger.

Usually there is not much sensitiveness in the parts, and it is sometimes possible to accomplish nearly as much tinting as is needed, over a considerable space, at a single sitting. In other more sensitive subjects this cannot be done, and the tatooing must be repeated, perhaps three or four times, to obtain the desired result. Generally, so much can be gained at a single performance that a patient appreciates the resulting benefit and willingly consents to a repetition of the tatooing.

If possible, the use of forceps to hold the eyeball should be dispensed with. If used, the conjunctiva should be taken hold of above the cornea, so that, in case the coloring matter should reach the punctures made by the forceps, the stain which would result may be so placed as to be concealed by being covered by the upper lid.

This operation should not be performed in cases where we find only a recent opacity of the cornea, as in many of these instances we may expect a gradual absorption of the cloudiness, especially if the patient is young. Of course it is needless to resort to this means, intended mainly for cosmetic effect, where both eyes are sightless, unless in exceptionally great deformity. But, where one eye is perfect, the removal of a blemish from the other is often a great boon to sensitive individuals, especially to ladies.

Scarcely any precautions are needed after this little operation, and it may safely be repeated in a few days, sometimes in a day or two if desirable. It may be undertaken by any physician having a delicate touch, even if quite inexperienced in eye operations. It should not be done, however, while any lingering irritation of the internal parts of the eye is present. It is especially adapted to cases of dense opacity, either partial or affecting the whole extent of the cornea. It may also be resorted to with advantage in some special conditions, where the cornea is transparent, but where it is desirable virtually to limit the area of the pupil by excluding some of the pencils of the rays of light; as, for instance, in some cases where iridectomy has been done, or in conical cornea."—*Boston Med. and Surg. Journal.*

